# The Management of Preeclampsia: A Comprehensive Review of Current Practices and Future Directions

**DOI:** 10.7759/cureus.51512

**Published:** 2024-01-02

**Authors:** Dhruvikumari D Sharma, Nidhi R Chandresh, Ayesha Javed, Peter Girgis, Madiha Zeeshan, Syeda Simrah Fatima, Taneen T Arab, Sreeja Gopidasan, Vineesha Chowdary Daddala, Kalgi V Vaghasiya, Ameena Soofia, Maneeth Mylavarapu

**Affiliations:** 1 Biochemistry, Spartan Health Sciences University, Vieux Fort, LCA; 2 Medicine, Avalon University School of Medicine, Willemstad, CUW; 3 Internal Medicine, Army College of Medical Sciences, New Delhi, IND; 4 Gynecology, Hearts International Hospital, Rawalpindi, Rawalpindi, PAK; 5 Internal Medicine, Ross University School of Medicine, Bridgetown, BRB; 6 Internal Medicine, Fatima Jinnah Medical University, Lahore, PAK; 7 Internal Medicine, Rajarajeswari Medical College and Hospital, Bangalore, IND; 8 Family Medicine, Saint James School of Medicine, Chicago, USA; 9 Internal Medicine, American International School of Medicine, George Town, GUY; 10 Medicine, Kamineni Institute of Medical Sciences, Narketpally, IND; 11 College of Medicine, Community Health Center (CHC) Vartej, Vartej, IND; 12 Internal Medicine, Shadan Institute of Medical Sciences, Hyderabad, IND; 13 Public Health, Adelphi University, Garden City, USA

**Keywords:** emerging therapies, management protocols, pregnancy complications, hypertensive disorders of pregnancy, preeclampsia

## Abstract

Preeclampsia (PE) is a disease in pregnancy that is characterized by new-onset hypertension end-organ dysfunction, often occurring after 20 weeks of gestation. Risk factors include a prior history of PE, diabetes, kidney disease, obesity, and high maternal age at pregnancy. Current treatment and management guidelines focus on the management of high blood pressure and any potential complications. The only known curative treatment is termination of pregnancy (either induction of delivery or cesarean section). However, the current guidelines and recommendations lack adequate prediction markers and are unable to prevent maternal and fetal mortality. There also exists a need for multidisciplinary collaborative action in view of the quality of life and psycho-educational counseling.

## Introduction and background

‘Preeclampsia’ (PE) as a disease refers to new onset hypertension with significant end-organ dysfunction in the presence or absence of proteinuria in a pre-gestational normotensive patient, typically after 20 weeks of gestation [1]. PE accounts for almost 8% of all gestational-related complications and excess of greater than 50,000 maternal deaths and 500,000 fetal deaths globally [2]. Over the years, significant progress has been achieved in understanding the risk factors and pathophysiology of PE. However, in terms of diagnosis and management, the existing guidelines may not be sufficient to reduce maternal and fetal mortality effectively. This review outlines the pathophysiology, current diagnosis and management guidelines, challenges in the current guidelines, and future directions.

## Review

I. Pathophysiology of PE

PE is a disorder of pregnancy that typically presents after 20 weeks of gestation, with its core clinical manifestation of hypertension. This pregnancy-specific complication is known to cause significant perinatal and maternal morbidity and mortality. PE clinical presentation is variable from one patient to another. One way PE can present in a patient is with a new onset of hypertension and proteinuria [1]. PE can also present with new-onset hypertension in addition to end-organ dysfunction in the absence of proteinuria [1]. Hypertension in the setting of PE is defined as systolic blood pressure (SBP) ≥140 mm Hg and diastolic blood pressure (DBP) ≥ 90 mm Hg in a previously normotensive pre-gestational female. Proteinuria in the setting of PE is defined as protein ≥300 mg in a 24-hour urine collection [2].

Risk factors for the development of PE can be attributed to a number of factors. First and foremost, a prior history of PE significantly increases the risk for the development of PE in subsequent pregnancies. Pregestational diabetes, antiphospholipid syndrome, obesity, and chronic hypertension are all risks for the development of PE. In addition, advanced maternal age, history of chronic kidney disease, obesity, and nulliparity also increase the risk of development of PE [2]. Genetic predisposition is associated with an increased risk of PE, and incidence is increased in African-American women. Additionally, individuals born from mothers who had PE during pregnancy also increased the risk. In more recent studies, a genome-wide association study (GWAS) specifically studied chromosome 13, which contains a single-nucleotide polymorphism near the FLT1 locus and was found to increase the development of PE. FLT1 is an fms-like tyrosine kinase-1 (sFlt1) anti-angiogenic factor that has been shown to antagonize placental growth factor as well as vascular endothelial growth factor (VEGF), leading to endothelial dysfunction [3]. Therefore, the study indicated that Trisomy 13 was associated with higher levels of FLT1 and, therefore, an increased risk of developing PE [4].

The pathogenesis of PE is rather complex. However, maternal endothelial dysfunction due to placental factors plays a crucial role in its pathogenesis. PE is generally characterized by abnormal remodeling of spiral arteries, defective placentation, placental ischemia, oxidative stress of the maternal-fetal interface, and angiogenic disequilibrium within the maternal circulation, leading to end-organ dysfunction. The proposed two-stage theory of the pathophysiology of PE has widely been accepted. Maternal risk factors that make individuals more susceptible can influence the inadequate and dysfunctional invasion of cytotrophoblasts into the spiral arteries, leading to abnormal placentation, known as the first stage. This leads to a reduction in perfusion of the placenta, resulting in the release of anti-angiogenic factors and additionally results in endothelial and systemic vascular dysfunction, known as the second stage [5].

PE is associated with inadequate spiral artery remodeling, characterized by failure of trophoblasts to appropriately remodel the spiral arteries from low flow, high resistance to the large diameter, and low resistance vessels necessary to sustain pregnancy. Furthermore, this leads to placental under-perfusion, hypoxia, and ischemia, which aids in the stimulation of the placental release of angiogenic substances. As a result, this causes widespread maternal systemic endothelial dysfunction, leading to the formation of hypertension, with or without proteinuria. In addition, this also leads to many other clinical manifestations associated with PE, such as headache, nausea, vomiting, abdominal pain, and visual disturbances.

Normally, in the process of placental implantation, cytotrophoblasts invade the spiral arteries and form vascular sinuses that provide nutrition to the growing fetus. In an otherwise normal pregnancy, invasion of the spiral arteries leads to extensive remodeling of maternal spiral arteries, resulting in the formation of high-flow vessels [6]. However, in the development of PE, the failure of cytotrophoblasts to adequately transform from the proliferative epithelial subtype into the invasive endothelial subtype results in inappropriate remodeling of spiral arteries. Furthermore, this inadequate formation of spiral arterioles causes them to become narrow in shape and results in placental ischemia [7]. Placental hypoperfusion, therefore, stimulates the release of vasculogenic and angiogenic factors that disrupt the maternal endothelium, altering vascular tone and permeability.

PE is also characterized by oxidative distress. Due to poor spiral artery invasion, hypoxia itself leads to oxidative stress with increased expression of reactive oxygen species (ROS). In addition, oxidative stress leads to the formation of anti-angiogenic factors [8]. Accumulation of advanced oxidation protein products (AOPPs) leads to alternations in the regulation and signaling of angiogenic pathways and, therefore, aids in the development of PE [8].

The endothelial dysfunction and overall systemic vascular dysfunction are shown to be related to the enhanced production of placental antiangiogenic factors. The VEGF and placental growth factors get antagonized by soluble fms-like tyrosine kinase 1 (a placental antiangiogenic factor), which leads to the deterioration of endothelial dysfunction. This event leads to an increase in hypertension and proteinuria, both found in pre-eclamptic patients. The endothelial dysfunction also causes other known damages to the organs of the carrier of the placenta due to the endothelium controlling the smooth muscle tone and the regulation of anti-coagulant and platelet functions. Venous congestion is also seen in the downfall of endothelial dysfunction as it reduces the blood flow to the organs, including but not limited to the heart [8].

Classification of a disease into subtypes can be done along various dimensions, such as epidemiological variables like timing of onset, presentation, and outcomes. In PE, studies report multiple subtypes possibly leading to a common presentation rather than one common pathogenesis. This could be one of the reasons substantial success in managing and preventing PE was not achieved [9].

II. Current management protocols

A. Anti-hypertensive Agents

The current anti-hypertensive treatment guidelines in PE suggest methyldopa (0.5-3 gm/day orally in divided doses) as the drug of choice. The next best alternative is labetalol (0.2-1.2 gm/day per orally in divided doses), slow-release nifedipine (10-30mg per orally), hydralazine (5mg IV given slowly over 1-2 min, 30-90 mg once a day). The second-line agents include clonidine (0.1-0.6 mg/day in divided doses), hydrochlorothiazide (12.5-25 mg/day per orally), nicardipine (3-9 mg/hour IV), sodium nitroprusside (0.24-5 μg/kg/min) [10]. Other drugs that can be used as alternatives are verapamil, diazoxide, prazosin, and oxprenolol.

Methyldopa acts at the central alpha-adrenergic receptor and stimulates it, resulting in a lowering of the sympathetic outflow of noradrenaline. Labetalol effectively blocks both alpha- and beta-adrenergic receptors and demonstrates a rapid onset of action in comparison with methyldopa. Nifedipine, a calcium channel blocker, is preferred in a slow-release or long-acting form over the short-acting form as the short-acting form may reduce uteroplacental blood flow. Hydralazine is a potent direct vasodilator that is mostly used in hypertensive emergencies or as an alternative in refractory hypertension. It is also important to note that potent antihypertensives like angiotensin-converting enzyme (ACE) inhibitors, angiotensin receptor blockers (ARBs), and spironolactone are not used (contraindicated) during pregnancy due to their teratogenic effects [11]. The NHBPEP (National High Blood Pressure Education Program) also advises slow-release nifedipine, beta-blockers (other than atenolol), and a diuretic as alternatives [12].

B. Magnesium Sulfate

Eclampsia (seizure) is a common complication of PE, and antihypertensive agents alone cannot prevent the development of eclampsia. Hence, magnesium sulfate is used owing to its neuroprotective action. While magnesium sulfate is not typically recommended for use as an antihypertensive agent, it is used routinely to prevent the development of seizures in pregnant women with severe features of PE. The American College of Obstetricians and Gynecologists (ACOG) recommends restricting the use of magnesium sulfate to PE with severe features. The criteria include SBP of 160 mm Hg or higher or DBP of 110 mm Hg or higher on at least two occasions four hours apart, thrombocytopenia, impaired LFTs (liver function tests), pulmonary edema, new onset headache unresponsive to medications, and visual disturbances [13]. Magnesium sulfate acts on the acetylcholine receptors, N-methyl-D-aspartate (NMDA) receptors, and calcium channels in the central nervous system (CNS). Magnesium sulfate also protects the blood-brain barrier and reduces the formation of cerebral edema [14]. Furthermore, it also acts as a vasodilator in the cerebral and peripheral vasculature. Magnesium sulfate is generally considered superior to other anti-convulsants like phenytoin, diazepam, and nimodipine.

The current management guidelines suggest a loading dose of 4-6 gm of magnesium sulfate, administered via infusion pump over 20-30 minutes, followed by a maintenance dose of 1-2 gm per hour as continuous IV infusion, continued until 24 hours after the delivery. Monitoring is required when magnesium sulfate is being administered due to toxicity and adverse effects. Hypermagnesemia or magnesium toxicity causes areflexia (loss of reflexes, particularly patellar deep tendon reflex) at 8-10 mEq/L of blood magnesium levels and respiratory paralysis at > 13 mEq/L of magnesium levels. Further higher levels may lead to cardiac arrest [14].

C. Delivery: Timing and Methods

The only curative treatment available for PE is termination of pregnancy, whether it be induction of labor or cesarean section. Induced delivery is indicated at 37 weeks of gestation at the earliest. According to ACOG Preeclampsia Guidelines, two approaches are recommended for pregnant women with PE, based on the gestational age (34 weeks 00 days) [15]. Expectant management is recommended if the gestational age is less than 34 weeks [16]. However, in the presence of severe features, expectant management provides benefits to the fetus/newborn but carries potential risks to the mother [16]. Regarding delivery, the factors that make a pregnant woman with PE - a candidate for delivery are outlined in Table 1 [13,15,16].

**Table 1:** Candidate for Delivery in Preeclampsia HELLP: Hemolysis, Elevated Liver enzyme levels, and Low Platelet levels; RUQ: Right upper quadrant; MI: myocardial infarction; RFTS: renal function tests; UA - REDF: umbilical artery - reversed end-diastolic flow; CR.: creatinine Table Credits: Kalgi V. Vaghasiya

Maternal Factors	Fetal Factors
Severe hypertension, which is unresponsive to an antihypertensive agent	Abnormal antenatal testing
Complaints of persistent headache or persistent RUQ/epigastric pain, unresponsive to the treatment	Fetal demise
Complaints or findings of visual disturbance or altered sensorium or motor deficit	Fetal lethal anomaly or extreme prematurity
Diagnosis of Stroke or MI	Doppler: UA - REDF
Diagnosis of HELLP syndrome	-
Worsening RFTs (Serum Cr. > 1.1)	-
Pulmonary edema	-
Eclampsia	-
Placental abruption or bleeding in the absence of placenta previa	-

Once the decision on delivery is reached, the mode of delivery is another concern. Regarding whether to induce delivery or perform a cesarean section, a cervical score is one of the essential factors to be considered. Induction of labor in patients with poor cervical scores has been found to be associated with failed induction, prolonged labor, and a high rate of conversation to cesarean section [15]. Induction or augmentation of labor is favorable when the patient has a clinically stable condition with a favorable cervix and normal growth of the fetus. Induction of labor should be considered a reasonable option in patients with severe PE at ≤ 34 weeks' gestation and has a better chance of having a successful normal vaginal delivery. The Bishop score upon admission serves as the best indicator of a successful delivery. However, the probability of a successful induction increases with advancing gestational age [17]. Strong evidence suggests that opting for a scheduled delivery reduces the risk of maternal complications and severe hypertension when compared to waiting and managing the situation expectantly. However, there is an increase in neonatal unit admissions due to premature births in planned deliveries, but there is no evidence to suggest a higher rate of neonatal health issues in such cases [16].

III. Challenges in diagnosis and current treatment modalities

PE contributes considerably to maternal morbidity and mortality worldwide. Predicting, diagnosing, and managing PE is indeed challenging, and while there have been advances in understanding the pathophysiology and underlying disease, current protocols do have some limitations [18]. One major limitation is the lack of effective predictive biomarkers that can reliably identify women at risk early in pregnancy. This makes it difficult to implement preventive measures or interventions before symptoms manifest. Additionally, there is a need for more accurate diagnostic tools to differentiate PE from other hypertensive disorders during pregnancy. The current diagnostic criteria rely entirely on clinical symptoms and signs, which may not be specific enough, leading to a spectrum of misdiagnosis, overdiagnosis, and underdiagnosis. Furthermore, existing treatment options for PE are limited, and the only definitive cure is delivery of the baby and placenta. The challenges in current diagnosis and management are outlined in Table 2.

**Table 2:** Challenges in Current Diagnosis and Management of Preeclampsia Table Credits: Maneeth Mylavarapu

S. No.	Factor	Limitations
Challenges in Current Diagnosis		
1.	Predictive Biomarkers	The identification of reliable early biomarkers for predicting preeclampsia is challenging. Many studies have explored various markers, such as angiogenic factors (like sFlt-1 and P1GF), but their sensitivity and specificity may vary, and no single biomarker has proven consistently reliable for predicting preeclampsia in all cases [19]. Another problem is using biomarkers too late or only utilizing them from symptomatic patients. Although analytical data has shown that biomarkers like placental growth factor assay and tyrosine kinase1 have shown excellent precision in diagnosing preeclampsia, there is limited use [20].
2.	Diagnostic Criteria	Diagnostic criteria, such as elevated blood pressure and proteinuria, lack specificity and may lead to overdiagnosis or underdiagnosis. Additionally, these criteria may not be sensitive enough to capture cases of atypical or early-onset preeclampsia [13].
Challenges in Current Management		
3.	Treatment Options	Limited pharmacological interventions exist for preeclampsia, and the only curative treatment is termination of pregnancy.
4.	Adverse Effects of Medications	Anti-hypertensives: Antihypertensive drugs, which are frequently prescribed for preeclampsia, might have negative side effects, including hypotension, dizziness, and electrolyte abnormalities [21]. Magnesium Sulfate: Although magnesium sulfate is used to prevent seizures in cases of severe preeclampsia, high dosages can cause respiratory depression and neuromuscular blockade [22].
5.	Induction of Labor	Induction of labor isn't always feasible. In some circumstances, like previous cesarean section, inducing labor might result in uterine rupture [23].
6.	Cesarean Section	Standard risks of any surgery, like infection, bleeding, and post-anesthetic complications, apply to cesarean section as well.
7.	Postpartum Complications	Complications from preeclampsia can persist even after delivery. Preeclamptic women are more likely to experience cardiovascular problems later in life [24].
8.	Neonatal Complications	The risk of neonatal problems such as respiratory distress syndrome and intraventricular hemorrhage is increased by preeclampsia-related premature delivery [25].

IV. Emerging treatment and preventive approaches

Multiple novel predictors, therapies, treatment, and monitoring delivery models are under various phases of development and clinical trials in diagnosing and managing PE.

A. Novel Drug Therapies

Statins play a role in the management of inflammation, and it is believed that the development of PE is caused by the worsening of inflammation. The mechanism by which statins suppress C-reactive protein is independent of their ability to inhibit HMG-CoA reductase activity. Statins have a beneficial impact on endothelial cells even when their dysfunction is severe. Their ability to increase nitric oxide (NO) levels and vascular relaxation is facilitated by their stimulation of endothelial nitric oxide synthase (eNOS) expression via the PI3k/Akt pathway or by upregulating haem-oxygenase-1 levels. Statins, particularly pravastatin, have been shown in studies to positively impact blood pressure and lower the likelihood of adverse pregnancy outcomes [26].

Because PE can be explained by abrupt atherosclerotic changes in the uterine arteries, pravastatin, by lowering the synthesis of cholesterol, could increase placental perfusion in the condition. Through overexpression of complement inhibitor decay-accelerating factor (DAF) and reduction of C5a activation in the cervix, pravastatin appears to lower complement activation in animal (mouse) models [27]. The American Food and Drug Administration (FDA) does not recommend the use of statins for all pregnant women despite the fact that they have been found to contribute to PE risk mitigation or stabilization of its clinical features. This is considering statins lower cholesterol levels, which decrease the fetus’s ability to absorb them, increasing the risk of miscarriages or fetal congenital defects [26].

Low-dose aspirin, given at 81-150 mg per day, is the most evidence-based preventive measure. It usually starts before 16 weeks of gestation [28]. Prophylactic administration of low-dose aspirin (60-150 mg) starting during the first trimester of pregnancy decreased the risk of PE and significantly unfavorable perinatal outcomes in women who were at elevated risk. More specifically, the data suggested slightly lower chances of intrauterine growth retardation (IUGR), preterm birth, and possibly perinatal mortality. There was also a notable variation in birth weight, which was consistent with a decreased risk of preterm birth and IUGR [29].

Across a clinically diverse set of trials, daily aspirin usage throughout pregnancy for those at elevated risk of PE consistently yielded positive benefits on perinatal mortality, preterm birth, fetal growth restriction, and PE diagnosis. A substantial amount of trial data indicates that there is no conclusive evidence of major risks related to daily low-dose aspirin use during the second and third trimesters of pregnancy [30]. The timing of initiation affects how well aspirin supplementation works. There is very minimal benefit to PE if it is started after 16 weeks of gestation [31].

B. Targeted Therapies

New approaches for diagnosis, prediction, and management may open up as the importance of angiogenic imbalance in PE becomes more apparent [32]. Several anti-angiogenic factors, including soluble Flt1 (sFlt1) and soluble endoglin (sEng), are produced by the placenta in larger quantities than normal in preeclamptic pregnancies [3]. There have also been recommendations for therapeutic approaches that target the angiogenic imbalance in PE. Treatment with VEGF-121 has been demonstrated to lower high blood pressure linked to placental ischemia and sFlt1 overexpression, improve glomerular filtration rate, and enhance endothelial function in an animal (rat) model [32].

C. Immunomodulatory Approaches

IL-17 serves as a pivotal mediator in inflammation and antibacterial responses. It has been associated with compromised tolerance in various clinical conditions, like PE, autoimmunity, contact dermatitis, and transplant rejection. Secukinumab, a monoclonal antibody targeting IL-17, has been employed to modify the Th imbalance in psoriasis, contact dermatitis, and discoid lupus erythematosus. Tibulizumab, a dual antagonist tetravalent antibody against IL-17 and B-cell activating factor (BAFF) used in the management of Sjogren's disorder, presents as a potential method to alleviate inflammation in PE that may be either causal or a downstream mediator. Other biologic agents, like TNF-α blockade, have proven successful in reducing inflammation in inflammatory bowel disease and have been safely utilized during pregnancy, showing promise in addressing the observed difference in Th1/Th2 levels in PE [33]. Eculizumab, a monoclonal antibody that blocks C5, has been approved by the FDA to be used in pregnancy with paroxysmal nocturnal hemoglobinuria and atypical hemolytic uremic syndrome. It has also demonstrated efficacy in treating HELLP syndrome, leading to improved laboratory parameters and extended pregnancy in a reported case. Zilucoplan, a small molecule inhibitor of C5a used for myasthenia gravis, may hold possible benefits in HELLP or PE treatment [33].

Hydroxychloroquine (HCQ), an immunomodulatory agent with anti-inflammatory and antimalarial properties, is frequently employed in treating autoimmune diseases, including those posing a risk for PE, such as lupus or antiphospholipid syndrome. HCQ inhibits NFĸB activity by blocking the phosphorylation of kappa B inhibitor, thereby downregulating inflammatory factors controlled by NFĸB. As a result of HCQ supplementation, cytotrophoblastic cells exhibit reduced sFlt1 secretion and increased proangiogenic factors [31]. Furthermore, HCQ's antithrombotic activity may prevent fibrin formation, mitigating the risk of placental insufficiency and PE development. Numerous studies suggest that HCQ holds promise against PE, with findings indicating higher rates of live births, lower pregnancy morbidity, and reduced likelihood of PE development [31].

D. Telemedicine and Remote Monitoring

ACOG classifies the telehealth models into three broad categories, namely, synchronous (real-time), asynchronous (sending medical imaging for later interpretation), or remote monitoring [34]. The COVID-19 pandemic accelerated the adoption of telehealth services for pregnant women, proving to be both safe and cost-effective. Telehealth, including text-based systems, has been piloted in the UK, demonstrating improved patient satisfaction, reduced visits to day-assessment units, and lower appointment costs [35]. While stringent feto-maternal surveillance is traditionally recommended through inpatient monitoring to avoid complications, post-COVID-19 telehealth programs have successfully delivered around 50% of antenatal consultations without compromising the diagnosis and management of common pregnancy complications associated with PE, compared to conventionally delivered antenatal care [36]. However, telehealth and remote monitoring do have exceptions since they cannot be utilized in severe PE.

V. Future directions and research

A. Ongoing Clinical Trials, Technologies, and Innovations

The management of PE is at a crossroads, requiring new perspectives and committed research efforts in the creation of cutting-edge biomarkers, targeted drug therapies, advanced diagnostic technologies for prompt identification, and efficient therapies to improve outcomes for both the mother and the fetus.

A noteworthy clinical trial conducted by Paidas et al. underscored the limited benefits of recombinant human antithrombin in preterm PE, as it failed to extend pregnancy duration or yield significant improvements in neonatal and maternal outcomes [37]. In a separate recent study, it was demonstrated that obese women with PE receiving a specific magnesium dosage regimen consistently achieved therapeutic blood magnesium concentrations, a crucial aspect of the prevention of eclampsia in preeclamptic patients [38]. Furthermore, Culebras et al. conducted a double-blinded, randomized, multicenter trial to evaluate the efficacy and side effects of intrathecal nalbuphine and intrathecal morphine for postoperative pain reduction following cesarean births. Remarkably, both options did not lead to respiratory depression in either the mother or the infant, with intrathecal nalbuphine at 0.8 mg proving highly effective for intraoperative and immediate postoperative pain relief. It's important to note that only morphine offered long-lasting analgesia [39].

As the field of diagnosis and management of PE advances, more trials are underway to further improve delivery timing and enhance fetal outcomes. For example, ongoing trials aim to evaluate whether Digibind® (anti-digoxin antibody) can prolong the timing of delivery in severe PE cases. This delay could provide maternally delivered steroids with additional time to mitigate respiratory difficulties in preterm newborns. These innovative investigations hold the promise of revolutionizing PE care, offering new hope for expectant mothers and their fetuses [40]. Moreover, other researchers are dedicated to mitigating postpartum hypertension in preeclamptic women by employing loop diuretics like torsemide. These diuretics accelerate the removal of excess fluids that accumulate due to PE, reducing the incidence of postpartum hypertension [41]. However, diuretics could not help with other subtypes of PE or gestational hypertension since the use of diuretics during pregnancy will increase the risk of pre-term delivery [42].

B. Multidisciplinary Collaboration in Research on PE

Pregnancy is an emotional crisis for a lot of women, leading to stress and anxiety. It is due to many stress factors, one of them being PE. It is an independent stress factor. After the diagnosis of this condition, immediate and appropriate treatment and examination of the mother are of utmost importance [43]. The WHO definition of quality-of-life states “the perception a person has of their own life within the context of their culture and values and personal objectives, standards, and concerns.” During pregnancy, the mother’s own health standards and life’s perception change as the focus moves on the fetus during this time. Therefore, women undergo psychological adaptations because of concerns and fears related to pregnancy, which might affect their quality of life [44]. PE can lead to extended in-patient hospital stays for diagnosis, treatment, or multiple follow-ups of the patients, and there is a possible occurrence of unforeseeable events, e.g., preterm labor and fetal complications, thus causing a major strain on pregnant women [43]. Additionally, there are unexpected medical interventions required, and sometimes fear of death can also lead to anxiety and severe fright in mothers. The mean anxiety scores are seen to be higher in women diagnosed with PE. Rigó et al. reported remarkably high anxiety levels in pregnant women with PE when compared to healthy pregnant women [45].

The primary objective in a woman with a prior history of PE is to reduce the incidence of modifiable risk factors for future recurrence. Thus, it is a must that maternal health be improved and optimized prior to conception. This can be achieved via psycho-educational counseling. It is recommended that it should begin six weeks postpartum in patients these patients. The recommendations include risk modification strategies to decrease recurrence, i.e., strict monitoring of blood pressure, lifestyle improvement and modifications, and glycemic control should be attempted [46]. Crovetto et al. reported that the Mediterranean diet and mindfulness help to reduce the incidence of small of age birth weight in newborns [47]. Thus, providing mental health interventions for a pregnant woman is essential to provide security for the emotional development of children. Hence, taking care of women with determinants of anxiety, as well as psychological counseling and timely referral to better and advanced diagnostic and treatment centers, can improve the quality of life and reduce maternal and fetal mortality and morbidity [43]. Women having low support from partners, family, and friends tend to have ineffective psychosocial resources, especially social stability and social participation, and thus receive insufficient emotional and psychological support from their social circle [46].

PE is a disorder with a high incidence in low- and middle-income countries compared to high-income countries [48]. The high prevalence of PE in these low- and middle-income countries is challenging due to deficient resources that cause difficulty in the diagnosis and management of this condition. Existing sociocultural, economic, and geographic barriers further delay the appropriate treatment and limited access to emergency obstetric care, then worsen the already prevailing poor outcomes. Thus, addressing a single factor alone is not enough, and a multifaceted approach needs to be analyzed, developed, and implemented with customized strategies and actions that are contingent on improving PE outcomes [49].

Adequate supply of resources, proper training for healthcare providers, and easy access to appropriate healthcare are crucial factors in PE management [50]. The midwives play a strategic role as mental-physical supporters. They are responsible for relaxing mothers and reducing their anxiety. Midwives would know the warning signs and facilitate timely diagnosis and treatment [43]. The role of midwives and medical professionals in conducting routine medical examinations, such as timely blood pressure measurement and monitoring of proteinuria during prenatal visits, is of utmost importance in the early detection of PE. The most effective screening method for early detection of PE is for the midwives to identify high-risk women and initiate appropriate management promptly. The potential benefits of calcium supplementation, antioxidant use, and blood pressure regulation in reducing the risk of PE [50].

## Conclusions

In conclusion, PE is a complex hypertensive disorder of pregnancy characterized by abnormal placental and systemic vascular dysfunction. The current anti-hypertensive treatment guidelines recommend methyldopa as the drug of choice, and termination of pregnancy is the only curative treatment available. The current protocols have limitations, the major being the lack of effective predictive biomarkers that can predict the risk of PE before the onset of symptoms. The management of PE is at a crossroads, requiring new perspectives and committed research efforts in the creation of cutting-edge biomarkers, targeted drug therapies, advanced diagnostic technologies for prompt identification, and efficient treatments to improve outcomes for both the mother and the fetus. The multidisciplinary approach of pre-conceptional counseling in high-risk women, psychoeducational counseling of expectant mothers, and proper training of health care providers have significant roles in the reduction of maternal mortality and better outcomes.
